# A methodological exploration of distinguishing hair quality based on hair proteomics

**DOI:** 10.1186/s12953-024-00229-w

**Published:** 2024-05-02

**Authors:** Xiaolin Wu, Tao Zhang, Mingsong Mao, Yali Zhang, Zhenpeng Zhang, Ping Xu

**Affiliations:** 1 School of Medicine, Guizhou University, Guiyang, 550025 Guizhou China; 2 State Key Laboratory of Proteomics, National Center for Protein Sciences (Beijing), Beijing Proteome Research Center, Beijing Institute of Lifeomics, Beijing, 102206 China; 3 Research Unit of Proteomics & Research and Development of New Drug, Chinese Academy of Medical Sciences, Beijing, 102206 China; 4 Anhui Medical University, Hefei, 230022 China

**Keywords:** Hair, Proteomics, Keratin, Keratin-associated protein, Bleaching damage

## Abstract

Hair is an advantageous biological sample due to its recordable, collectable, and storable nature. Hair's primary components are keratin and keratin-associated proteins. Owing to its abundance of cystine, keratin possesses impressive mechanical strength and chemical stability, formed by creating disulfide bonds as crosslinks within the protein peptide chain. Furthermore, keratin is cross-linked with keratin-associated proteins to create a complex network structure that provides the hair with strength and rigidity. Protein extraction serves as the foundation for hair analysis research. Bleaching hair causes damage to the structure between keratin and keratin-associated proteins, resulting in texture issues and hair breakage. This article outlines various physical treatment methods and lysate analysis that enhance the efficiency of hair protein extraction. The PLEE method achieves a three-fold increase in hair protein extraction efficiency when using a lysis solution containing SDS and combining high temperatures with intense shaking, compared to previous methods found in literature. We utilized the PLEE method to extract hair from both normal and damaged groups. Normal samples identified 156–157 proteins, including 51 keratin and keratin-associated proteins. The damaged group consisted of 155–158 identified proteins, of which 48–50 were keratin and keratin-associated proteins. Bleaching did not cause any notable difference in the protein identification of hair. However, it did reduce coverage of keratin and keratin-associated proteins significantly. Our hair protein extraction method provides extensive coverage of the hair proteome. Our findings indicate that bleaching damage results in subpar hair quality due to reduced coverage of protein primary sequences in keratin and keratin-associated proteins.

## Introduction

Hair is a type of epidermal appendage found on the human body that showcases a complex, structural composition [1]. Hair contains large amounts of protein, small quantities of water, and trace amounts of lipids, pigments, and metal elements [2]. Protein makes up 60–95% of hair's overall chemical composition. A cross-linked structure composed of keratin and keratin-associated protein (KAP) maintains the chemical and mechanical stability of hair [3]. Hair's distinctive proteins and structures provide exceptional stability in a normothermic environment [4].

When viewed under an optical microscope, hair can be divided into three layers: Cuticle, Cortex, and Medulla [5]. The Cuticle, located on the outermost layer of hair, serves as a protective layer. Hard keratin comprises the bulk of the Cuticle's composition, and due to this layer's weak resistance to abrasion, it is prone to damage and loss from scratching or bleaching, leading to hair that is brittle and rough [2, 6–8]. The Cortex, comprising 45% of the overall hair shaft, plays a crucial role in preserving moisture, determining hair thickness, curvature, and color [9–11]. Located at the hair's center, the Medulla replaces disulfide bonds with polypeptide bonds to maintain structural integrity. This results in Medulla proteins that are water-insoluble and challenging to separate [12–14].

The amorphous matrix and intermediate filaments composed mostly of keratin and keratin-associated protein are the foundational components of hair. High cross-linking between these proteins creates a complex network structure, making hair rigid and tough [15]. In human hair, protein characteristics determine that keratin mainly comprises acidic keratin (K31-K38) and basic-neutral keratin (K81-K86) [16, 17].

In 2013, Mellacheruvu et al. [18] developed a contaminant affinity purification mass spectrometry library (CRAPome) mass spectrometry data showing the presence of keratin as a contaminant in more than 90% of experiments. As keratin can potentially mask the signal of some clinically relevant markers of low abundance, it is often used as a potential contaminant library in proteome analysis [19]. However, it is important to note that keratin and other proteins in hair, as well as endogenous and exogenous substances, provide convenience and valuable human information for evidence-based use in modern medicine. Keratin isoform variations are a potential biomarker that can be used to characterize epithelial diseases, as indicated by the results of Karantza [20]. This opens up new paths for detecting new disease markers using hair proteome. This offers promising avenues for detecting new disease markers using hair proteome, thus identifying human characteristics and tracking human health effectively.

Hair has become an increasingly used sample for studying potential markers in diagnostic, therapeutic, and forensic toxicology disciplines over recent years. It offers the advantages of being easily recorded, collected, transported, and stored, as well as being cost-effective compared to conventional samples such as blood and urine [21–23]. However, the interlinking of keratin and keratin-associated protein within hair makes hair more difficult to extract than conventional tissue proteins or body fluid proteins. We optimized the extraction process of proteins from hair by changing lysis solution and treatment method, as well as by combining enzyme digestion and increasing the amount of digestive enzymes to enhance the efficiency of hair protease digestion. Pre-developed methods in our laboratory were used to perform proteomic analysis between normal and bleach-damaged samples, indicating that damaged samples showed some degree of protein loss, particularly keratin. We observed these changes might be due to the downregulation of protein coverage.

## Materials and methods

### Hair samples

The Xu laboratory supplied hair samples from two volunteers. The black hair sample was categorized as the 'normal' group whereas the yellow hair sample was categorized as the 'damaged' group. The damaged group reacted with normal hair in a solution of 1% ammonia + 2% hydroperoxide pH10 for 30 min to simulate bleaching damage.

### Hair whole protein extraction method

Ten hairs, measuring 1 cm in length, were pooled together and cut into 1 mm-long pieces in preparation for protein extraction. The sectioned hair was washed thrice with 20% methanol.

Six protein extraction methods were compared to establish their efficiency for human hair samples.

(I) GH method: After adding 240 μL of lysis buffer to the cleaned and sliced hair sample, homogenization of the hair fragments was done using a glass homogenizer (Kimble, Manzanillo, Mexico) (GH) until there were no visible fiber particles.
(II) LN-BH method: Hair fragments were initially ground with liquid nitrogen (LN), then homogenized using Silica beads (BioSpec, Bartlesville, USA) (BH) on a vortex machine. Vortexing was conducted for 30 s, followed by resting on ice for another 30 s. This process was repeated six times for a total duration of 6 min.
(III) RTO method: The protein extraction process used dried hair fragments with the addition of 240 μL of lysis buffer. The mixture was then shaken at 1200 rpm for 18 h at room temperature to extract the proteins.
(IV) LN-H95 method: The hair samples were ground into a fine powder using liquid nitrogen and a mortar. Then, 240 μL of Urea buffer was added, and the mixture was heated at exactly 95 °C for 5 min.
(V) SH65 method: The proteins were extracted from the dried hair fragments by the addition of 240 μL of lysis buffer, followed by shaking the mixture at 1200 rpm at a consistent heat of 65 °C (SH65) for 12 h.
(VI) H-90 + SH-65 method: Prior to heating at 90 °C for 5 h, a clean and sliced hair sample was dissolved in 120 μL of lysis buffer. After precipitation at 14,000 g for 1 min, the supernatant was gathered as fraction 1 (F1). Furthermore, the remaining hair pellet was mixed with an extra 120 μL of lysis buffer, and the subsequent protein extraction was done by shaking it for 12 h at 1200 rpm and 65 °C. The resultant supernatant was gathered as fraction 2 (F2). The combination of F1 and F2 was then collected as the entire protein for the H-90 + SH-65 proteome sample.

The Mase method [24]: The proteins were extracted from the dried hair fragments using 240 μL of lysis buffer. Subsequently, the protein was extracted by shaking the mixture at 1200 rpm and 35 °C for a length of 18 h, following the protocol outlined.

### Protein quality control analysis

The integrity of the extracted hair proteome was evaluated using a Coomassie-stained SDS gel. The protein was quantified using a brief SDS-PAGE electrophoresis procedure and the ImageJ software (https://imagej.net/) in compliance with the procedure described in [25].

### Proteomics sample preparation

The protein sample was iodoacetylated with 10 mM dithiothreitol (DTT) at 80 °C for 10 min followed by alkylation with 20 mM iodoacetamide (IAA) at room temperature for 30 min in the dark. The alkylated protein samples were separated with a 10% SDS-PAGE. After staining, the gel pieces containing proteins were cut into micelles of about 1 mm^3^. The gel was destained and dried. The proteins were In-Gel digested with 20 ng/μL of home-made Atrypsin [26] and home-made Lys-C [27](2:1). All tryptic peptides were extracted and dried before LC–MS.

### Protein identification by LC/MS/MS

The tryptic peptides were resuspended with 10 μL of sample loading buffer (0.1% FA in ddH_2_O). The concentration of peptides was determined by a Nanodrop one (Thermo Fisher Scientific, Palo Alto, CA).

The samples were injected into a UPLC EASY-nLC 1200 system. (Thermo Fisher Scientific, Palo Alto, CA) equipped with a self-packed capillary column (75 μm i.d. × 15 cm, 1.9 μm C18 reverse-phase fused-silica). In brief, the peptides were loaded onto an analytical column by an autosampler and eluted with a 60 min nonlinear gradient: 4–8% solvent B (0.1% formic acid in ACN) for 6 min, 8 − 26% for 6 min, 26 − 43% for 34 min, 26 − 43% for 15 min, 43 − 80% for 1 min, and finally holding at 80% for the last 4 min. The eluted peptides were analyzed online by an LTQ Orbitrap Velos (Thermo Fisher Scientific, Palo Alto, CA) with a data-dependent acquisition (DDA) mode. The chromatographed peptides were ionized under a high voltage (2 kV). The full scan was acquired from m/z 300 to 1600 with a resolution of 30 000 at m/z 200. The automatic gain control (AGC) was set to 1 × 106, and the maximum ion injection time (MIT) was set to 50 ms. The top 20 most intense peptide ions were subjected to further fragmentation in DDA mode via collision-Induced dissociation (CID) with 35% normalized collision energy and a target value of 5 × 10^4^ for AGC. Dynamic exclusion was set to 50 s to avoid redundancy detection.

### Proteomics data processing

All of the raw files were searched by MaxQuant (version 1.6.17) against the protein FASTA file from the UniProt database. For protein identification, specific digestion of trypsin was used with a maximum of two missed cleavages allowed. A minimal peptide length was set to seven amino acids. The precursor mass tolerance was 20 ppm, and the MS/MS mass tolerance was 0.5 Da. Cysteine carbamidomethylation was set as fixed modifications, while methionine oxidation and N-terminal acetylation were set as variable modifications.

### Bioinformatics analysis of identified peptides and proteins

Statistical analysis of quantitative mass spectrometry-based proteomic experiments was performed for all samples with Perseus (version 1.6.6.0) [28]. The GO and KEGG analyses were performed with Database for Annotation, Visualization, and Integrated Discovery (DAVID) (http://david.abcc.ncifcrf.gov/) [29].

### Data access

The mass spectrometry proteomics data for hair proteome have been uploaded to the ProteomeXchange Consortium website (http://proteomecentral.proteomexchange.org) via the iProX partner repository [30] with the dataset identifier IPX0005862001.

## Results

### Development of a highly efficient protein extraction method from hair

**Fig. 1:**
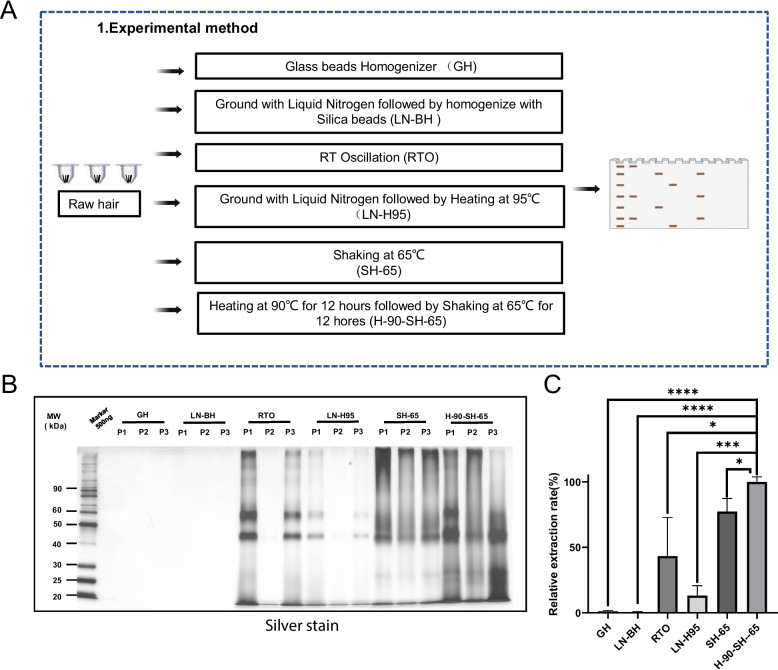
Development of a highly efficient protein extraction method from hair. **A** Workflow for the development of a highly efficient protein extraction method from hair. **B** Silver stained SDS-PAGE to compare the protein extraction efficiency among six methods. GH: Glass Homogenizer; LN-BH: Ground with Liquid Nitrogen (LN) followed by homogenize with Silica beads (BH); RTO: RT Oscillation; LN-H95: Ground with Liquid Nitrogen followed by Heating at 95℃; SH-65: Shaking at 65℃; H-90-SH-65: Heating at 90℃ for 12 h followed by Shaking at 65℃ for 12 h. **C** Quantitative analysis of the silver stained SDS-PAGE of B.* indicates *p* < 0.05. *** indicates *p* < 0.001. **** indicates *p* < 0.0001

To establish a highly efficient protein extraction method from a sample of human hair, we evaluated six different methods, as shown in Fig. 1A. The six methods compared overall protein extraction efficiency using three different hair fragmentation techniques and three different protein extraction methods. SDS-PAGE gels stained with Coomassie blue showed that the GH and LN-BH methods did not yield visible proteins (Fig. 1B and C). The RTO and LN-H95 methods yielded moderate amounts of protein with average extraction efficiency. However, these two methods were unstable, as only two of the three biological replicates showed visible protein. The SH-65 method extracted proteins from hair with higher efficiency and stability, as evident from highly consistent SDS-PAGE banding patterns across all three biological replicates. However, we observed that proteins with molecular weights under 40 kDa were almost entirely absent. The H-90-SH-65 method yielded the largest amounts of protein from hair, with highly reproducible, evenly distributed protein across all three experimental replicates (Fig. 1B). Quantitative results based on the stained SDS-PAGE gel showed that the average efficiency of the H-90-SH-65 method for extraction is 22.71% higher than the SH-65 method, and compared with other extraction methods, the efficiency is increased by at least 1.3 times (Fig. 1C). These results demonstrated that both strong shock and boiling can effectively improve the efficiency of protein extraction from hair.

### Development of high efficiency protein extraction buffer from hair

**Fig. 2:**
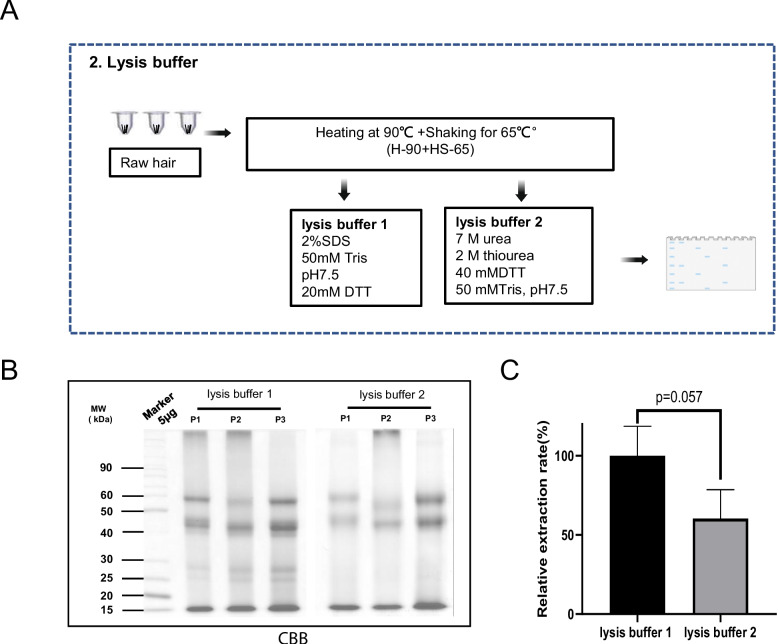
Development of high efficiency protein lysis buffer. **A** Workflow for the development of highly efficient protein lysis buffer from hair. **B** Coomassie brilliant blue stained SDS-PAGE to compare the proteins extracted with two lysis buffers. **C** Quantitative analysis of Coomassie blue stained SDS-PAGE of B

To investigate the impact of buffer components on the hair protein extraction efficiency, we compared the buffer solution containing a high concentration of SDS (lysis buffer 1) and high concentration of urea (lysis buffer 2) with the highest efficiency H-90-SH-65 extraction method (Fig. 2A). The gel electrophoresis results showed that SDS buffer produced more uniformly dispersed protein bands throughout the lane of the Coomassie brilliant blue stained gel compared to Urea buffer (Fig. 2B). The stained SDS-PAGE quantitative analysis revealed that protein extracted using the SDS buffer had 1.67 times higher yield than that of the Urea buffer (Fig. 2C). The effective extraction of proteins from hair can be achieved through combining long-term heating and strong impact with a simple high-concentration SDS lysis buffer. In comparing hair sample processing methods and optimizing buffer components, we determined that the combination of H-90-SH-65 and SDS-containing protein lysis solution demonstrated the highest extraction efficiency, and hence, we termed this technique the PLEE (PTM Lab for protein extraction from hair with high efficiency) method.

### Comparison of protein extraction efficiency between PLEE method and Mase method

**Fig. 3:**
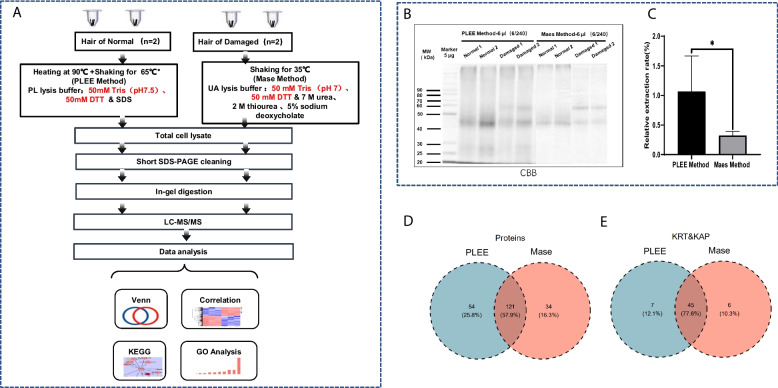
Comparison of protein extraction amount between two methods. **A** workflow comparing the efficiency of the PLEE method with the Mase method for the extraction of hair proteins. **B** Coomassie brilliant blue stained SDS-PAGE to compare the quantity and quality of proteins extracted by the two methods. **C** Quantitative analysis of Coomassie blue stained SDS-PAGE of **B**. **D** Venn diagram of the total number of protein identification by the two methods. **E** Venn diagram of the total number of KRT and KAP identification by the two methods. * indicates *p* < 0.05; KRT: keratin; KAP: keratin-associated proteins

**Fig. 4:**
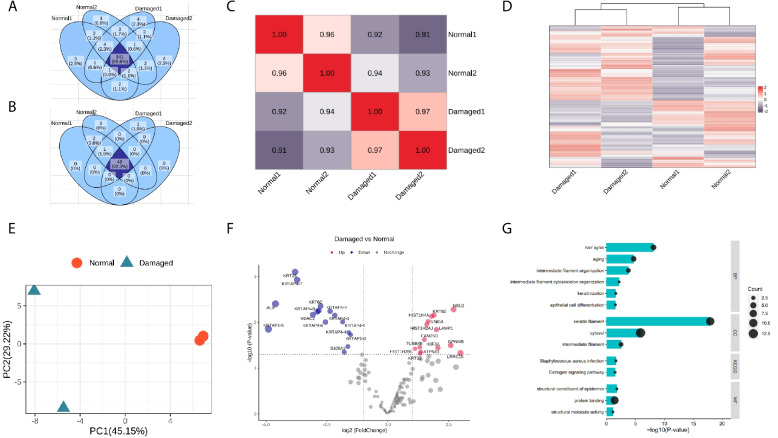
High-quality large-scale proteome based on the PLEE method characterizes hair injury by bleaching damage. **A** Venn diagram of 80.6% protein in the overlap of normal and damaged samples. **B** Venn diagram of 92.3% keratin and keratin-associated proteins in the overlap of normal and damaged. **C** The correlation among biological replicates and different conditions. **D** Cluster heatmap for damaged and normal groups; E. PCA analysis for damaged and normal groups. **F** Volcano plots showing the fold changes for damaged and normal data. The cut off were folded change > 2 and *p*-value < 0.05. **G** GO and KEGG term analysis of down-regulated proteins

**Fig. 5:**
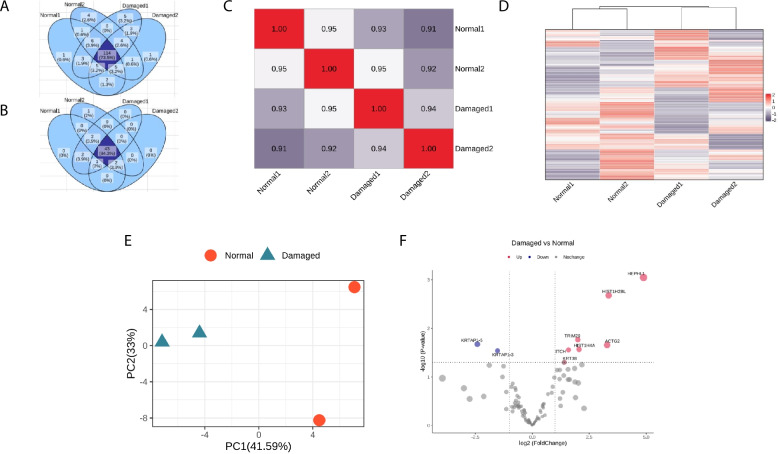
The resolution of which high-quality large-scale proteomics data used Mase method to distinguish samples is insufficient. **A** Venn diagram of 73.5% protein in the overlap of normal and damaged. **B** Venn diagram of 84.3% keratin and keratin-associated proteins in the overlap of normal and damaged. **C** The correlation among biological replicates and different conditions. **D** Cluster heatmap for damaged and normal groups. **E**. PCA analysis for damaged and normal groups. **F** Volcano plots showing the fold changes for damaged and normal data. The cut off were folded change > 2 and *p*-value < 0.05

To validate the protein extraction efficiency of the PLEE method for hair, we compared the efficiency of protein extraction of the PLEE method with the method reported by Mase laboratory (Mase method). To evaluate the efficiency of the two methods, we used normal hair and damaged hair for protein extraction (Fig. 3A). Equal amounts of protein were separated by 10% SDS-PAGE (Fig. 3B). The results showed that the PLEE method extracted significantly more hair protein than the Mase method, with a PLEE method protein extraction efficiency being 3.30 times that of the Mase method in both normal hair and damaged groups (Fig. 3C). Using consistent liquid phase and mass spectrometry conditions for both methods, peptide segment samples were prepared from an equivalent amount of protein, and proteomics data was obtained. The total number of identified proteins, keratin, and keratin-associated proteins in the two methods were compared. The results showed that the PLEE method identified 175 proteins, including 52 keratin-associated proteins and 51 keratin, while the Mase method identified 155 proteins, including 51 keratin-associated proteins and 50 keratin, respectively. Therefore, the PLEE method had a better total number of identified proteins (as indicated in Figs. 2, 3, 4 and 5 C-D), consistent with the SDS-PAGE observation. Using consistent liquid phase and mass spectrometry conditions for both methods, peptide segment samples were prepared from an equivalent amount of protein, and proteomics data was obtained. The total number of identified proteins, keratin, and keratin-associated proteins in the two methods were compared. The results showed that the PLEE method identified 175 proteins, including 52 keratin-associated proteins and 51 keratin, while the Mase method identified 155 proteins, including 51 keratin-associated proteins and 50 keratin, respectively. Therefore, the PLEE method had a better total number of identified proteins (as indicated in Figs. 2, 3, 4 and 5C-D), consistent with the SDS-PAGE observation (Fig. 3D-E).

### High-quality large-scale proteome based on the PLEE method.

We identified 155–158 proteins in both normal and damaged hair samples, of which 48–51 were keratin and keratin-associated proteins. Between the samples, 80.6% of total proteins and 92.3% of keratin and keratin-associated proteins overlapped (Fig. 4A-B). On average, 6 peptides were identified per protein (Table 1). The number of identified hair proteins was slightly different between normal and damaged hair samples. Figure 4C shows a high correlation between technical replicates, with a Pearson correlation of 0.96–0.97. In contrast, the correlation between different treatment groups was 0.91–0.94, which is lower than the correlations observed in both technical replicates.

**Table 1:** Results of proteomics database search

Sample	Total spectra	PSM	MS2 Identified (%)	Protein groups	Peptides	Full-cleavage (%)	KRT/KAP	Pep/Prot	PSM/Pep
Normal 1	57,442	7251	12.62	158	961	71.90	51	6.08	7.55
Normal 2	58,281	8011	13.75	156	975	72.10	51	6.25	8.22
Damaged 1	54,875	6499	11.84	157	909	70.52	50	5.79	7.15
Damaged 2	51,855	5891	11.36	155	864	72.11	48	5.57	6.82

Using principal component analysis and cluster analysis, we were able to distinguish between normal and damaged hair samples, which can be separated into two distinct groups (Fig. 4D-E). On the volcano plot in Fig. 4F, we filtered differentially expressed proteins between the two groups using a twofold change and p-value less than 0.05. We identified 16 down-regulated proteins, including 11 keratin and keratin-associated proteins, and 14 up-regulated proteins. The analysis of differentially expressed proteins revealed that the down-regulated proteins were enriched in various processes related to hair structure, such as the hair cycle, aging, intermediate filaments, keratinization, and others, which may contribute to the deterioration of hair quality caused by damage (Fig. 4G).

### The resolution of which high-quality large-scale proteomics data used Mase method

In normal and damaged hair samples, we identified 134–142 proteins, of which 48–52 keratin and keratin-associated proteins and 734–790 peptides. Between samples, 73.5% of the total protein and 84.3% of the keratin and keratin-associated proteins between the samples were overlapped (Fig. 5A-B). On average, 5 peptides were identified per protein (Table 2). The number of identified hair proteins between normal and damaged hairs was changed slightly. As shown in Fig. 5C, there is a high correlation between technique replicates, with Pearson correlation between 0.94–0.95. The correlation between different treatment groups was 0.91–0.95, which is lower than those in both technical replicates.

**Table 2:** Results of proteomics database search

Sample	Total spectra	PSM	MS2 Identified (%)	Protein groups	Peptides	Full-cleavage (%)	Keratin	Pep/Prot	PSM/Pep
Normal 1	51,545	5810	11.27	139	742	71.70	52	5.34	7.83
Normal 2	49,682	5395	10.86	134	740	71.76	49	5.52	7.29
Damaged 1	50,170	5765	11.49	142	790	73.16	50	5.56	7.30
Damaged 2	49,793	5080	10.20	137	734	73.30	48	5.36	6.92

The principal component analysis (PCA) and cluster analysis suggested that normal and damaged hair samples could not be clearly distinguished (Fig. 5D-E), and the entire sample set could be divided into two groups. We used a volcano plot to identify differentially expressed proteins between the two groups with a twofold change and p-value less than 0.05, resulting in the identification of 2 down-regulated keratin-associated proteins (KRTAP1-5 and KRTAP1-2) and 7 up-regulated proteins (as shown in Fig. 5F). Overall, our results indicate that the Mase method is inferior to the PLEE method in terms of the depth of protein identification and insufficient at distinguishing between samples.

### The protein sequence coverage of PLEE method was greater than that Mase method

**Fig. 6:**
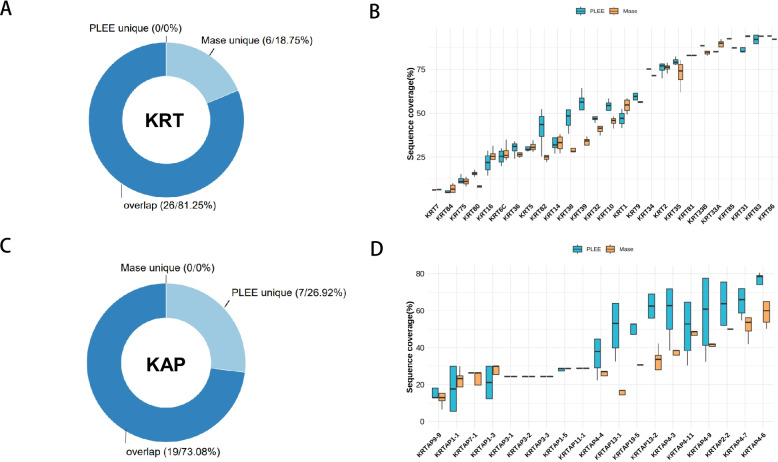
The sequence coverage of the identified proteins by the PLEE method wa s greater than that by the Mase method. **A** Donut chart of keratin identification by the two methods. **B** Boxplots of sequence coverage of keratin-associated proteins. **C** Donut chart of keratin-associated proteins identification by the two methods. **D** Boxplots of sequence coverage of keratin

**Fig. 7:**
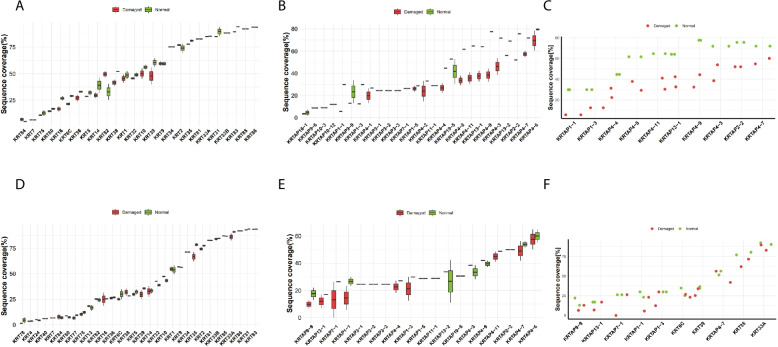
The sequence coverage of the identified proteins in the normal group was higher than that in the damaged group. **A** Boxplots of sequence coverage of keratin-associated proteins for the PLEE method. **B** Boxplots of sequence coverage of keratin for the PLEE method. **C** Scatter plot of sequence coverage distribution of TOP10 proteins. for the PLEE method. **D** Boxplots of sequence coverage of keratin-associated proteins for the Mase method. **E** Boxplots of sequence coverage of keratin for the Mase method. **F** Scatter plot of sequence coverage distribution of TOP10 proteins. for the Mase method

We compared the two hair protein extraction methods by analyzing the distribution of protein sequence coverage. Firstly, two methods jointly identified 26 keratins (Fig. 6A). The sequence coverage of 14 keratins by the PLEE method was greater than that by the Mase method (Fig. 6B). Second, t two methods jointly identified 19 keratin-associated proteins (Fig. 6C). The sequence coverage of 11 keratin-associated proteins in the PLEE method was greater than that in the Mase method (Fig. 6D). These results further supported that the PLEE method was more efficient than the Mase method.We compared the protein sequence coverage of normal and damaged hair samples using both PLEE and Mase methods. For PLEE, out of the 26 keratins and 26 keratin-associated proteins identified, the sequence coverage of 15 keratins and 16 keratin-associated proteins in the normal group was greater than that in the damaged group. We further plotted the 10 proteins with the highest difference and observed that the distribution of the normal group was more concentrated than that of the damaged group. Similarly, for Mase, out of the 32 keratins and 19 keratin-associated proteins identified, the sequence coverage of 9 keratins and 9 keratin-associated proteins in the normal group was greater than that in the damaged group (Fig. 7A-E). We also plotted the 10 proteins with the highest difference and observed a more concentrated distribution in the normal group than in the damaged group (Fig. 7F).

## Discussion

Hair samples bring forth multiple advantages over conventional samples, as they can be easily collected, stored stably over time, maintain a low probability of false positive results, and are non-invasively sampled [31]. The specific protein and structural composition of hair provide a stable medium at a standard temperature [32]. However, the unique protein and structural composition of the hair hinders efficient protein extraction through conventional methods.

We optimized the hair protein extraction method to improve the extraction efficiency. Following the comparison of several extraction methods and protein lysis buffers, we established the PLEE method, which demonstrated the highest protein extraction efficiency. The results clearly show that both strong shaking and prolonged boiling contribute to the efficient extraction of proteins from hair. Additionally, our experimentation with lysis buffer demonstrated that a high concentration of SDS lysis buffer effectively extracts protein from hair. In conclusion, the PLEE method exhibits superior efficiency in extracting proteins from hair compared to the Mase methods.

To comprehensively validate the extraction efficiency of our method, we conducted simultaneous hair protein extraction and proteomic analysis with methods from literature on damaged samples. As compared to the Mase method, the PLEE method identified a higher number of total and differential proteins in damaged samples. Additionally, the overlap of the two methods accounted for more than half, with each method having its distinct strengths. Furthermore, a majority of shared proteins had greater protein sequence coverage using the PLEE method compared to the Mase method, and the concentration and distribution of sequence coverage in the normal group of the PLEE method were more prominent than in the damaged group. These results suggest that the PLEE method has higher extraction efficiency, increased depth of coverage, better differentiation, and is more likely to identify specific mechanisms and homologous pathways.

The project conducted proteomic studies on damaged hair to explore the changes in hair protein composition and the consequences of damage. Prior research has indicated a significant difference in protein loss after hair damage [33, 34]. Our results also found that keratin and keratin-associated protein were downregulated in hair after damaged. On one hand, the gel plots revealed that healthy groups had greater protein extraction than the damaged ones for the same starting amount. On the other hand, protein analysis by enzymatic digestion for mass spectrometry showed a concentrated and highly distributed sequence coverage in healthy hair at the protein sequence level. In contrast, damaged hair protein sequences had varying degrees of loss and damage. The findings indicate that damage disrupts the internal structure of hair proteins, leading to a decrease in protein sequence coverage, which consequently affects the hair structure and causes further protein deletion.

In addition, in the differential analysis based on the PLEE approach, we screened some of the proteins that underwent down-regulation after damage, mostly keratin and keratin-associated proteins, including (KRT16, KRTAP4-7, KRTAP1-5, KRT6C, S100A3, etc.). This corresponds to changes in texture and shine of hair after damage.S100A3 is a calcium-binding protein, expressed primarily inside the hair microstructure of human hair, that plays an essential role in maintaining the structural integrity of hair fibers. Chemically-treated or UV irradiated hair can readily release S100A3 and cause modifications to the hair tissue structure [35, 36]. The enrichment analysis of the down-regulated proteins revealed that the damage mainly affected hair cycling, aging, and keratinization. From this, we can tentatively infer that the changes in hair phenotype under damage are mainly due to the loss of keratin, keratin-associated protein, or some key structural proteins such as S1003A, and the reduction of their protein sequence coverage. This in turn affects the stability of the internal structure of the hair, making most of the hair appear dry, frizzy, and lacking in shine after damage.

## Conclusions

Hair samples present several advantages over conventional samples due to their ease of collection, stable storage, low false positive probability, and non-invasive sampling. However, the unique protein and structural composition of hair hinders efficient protein extraction using conventional methods. The PLEE method was established as the most efficient method for protein extraction from hair, with strong shaking and prolonged boiling and a high concentration of SDS lysis buffer contributing to its efficiency. Compared to the Mase method, the PLEE method demonstrated higher extraction efficiency, increased depth of coverage, better differentiation, and a greater potential for identifying specific mechanisms and homologous pathways. Proteomic analysis using the PLEE method on damaged hair indicated that keratin and keratin-associated proteins were downregulated, leading to changes in hair texture, shine, and structure. Enrichment analysis revealed that damage primarily affected hair cycling, aging, and keratinization.

In conclusion, the results from PLEE-based hair proteomics data provide comprehensive biological insights into the changes in hair proteins caused by damage stimuli, identifying critical proteins needed for hair structure. Consequently, PLEE-based hair proteomics studies will prove to be immensely useful and powerful in the next generation assessment of hair health.

## Data Availability

Not applicable.
